# Salivary Chloride Intracellular Channel 1 (CLIC1) as a Hub of Gene-Gene Interactome of Periodontitis With Diabetes Mellitus

**DOI:** 10.7759/cureus.51877

**Published:** 2024-01-08

**Authors:** Shubhangini Chatterjee, Karthikeyan G, Pradeep Kumar Y

**Affiliations:** 1 Department of Periodontics, Saveetha Dental College and Hospital, Saveetha Institute of Medical and Technical Sciences, Saveetha University, Chennai, IND

**Keywords:** diabetes mellitus, biomarkers, network biology, saliva, periodontitis

## Abstract

Background and introduction

Periodontal disease is one of the most prevalent chronic conditions that affects the oral cavity. Identifying and predicting biomarkers is essential for the prevention of high-morbidity oral diseases. The genomic interaction network identifies common hub genes involved in crucial protein formation in periodontal inflammation. Diabetes mellitus is a metabolic disorder that has a double-edged sword relationship with periodontitis. Chloride intracellular channel 1 (CLIC1) was identified as a hub gene linking the pathogenesis of periodontitis and diabetes mellitus using a bioinformatic tool. Therefore, this current study aimed to assess the concentration of the pro-inflammatory biomarker CLIC1 in saliva among individuals with periodontal health and those with periodontal disease linked to diabetes mellitus.

Materials and methods

Differentially expressed genes (DEGs) in periodontitis were identified using datasets retrieved from the Gene Expression Omnibus (GEO) database. DEGs were combined to build the network, and GeneMANIA was used to find and rank the interconnecting genes. CLIC1 was identified as the hub gene, and clinical validation was done using patient samples. The study involved 30 participants. Based on clinical and radiographic periodontal findings, they were split into three groups: healthy (group 1, n=10), with periodontitis but no diabetes mellitus (group 2, n=10), and with periodontitis and diabetes mellitus (group 3, n=10). The collection of saliva samples, followed by quantifying these samples, was performed using an enzyme-linked immunosorbent assay (ELISA).

Results

From network graph analysis, it was discovered that CLIC1 functions as a hub gene in the majority of toll-like receptor pathways. The mean concentration of CLIC1 in saliva increased consistently as the disease was observed in periodontitis patients and periodontitis patients with diabetes mellitus.

Conclusion

CLIC1 concentrations were positively correlated with periodontitis in individuals with diabetes. Therefore, CLIC1 could be a diagnostic biomarker for patients with periodontitis. However, large-scale studies are needed to confirm more positive associations.

## Introduction

Periodontitis is the most typical form of inflammatory bone loss in people. It develops due to the inflammatory response to bacteria in the gingival crevicular area [1]. The multifactorial nature of periodontal disease arises from the involvement of multiple risk factors [2]. The primary goals of periodontal therapy are to establish a new attachment on a periodontal diseased root surface and regenerate all lost periodontal tissues [3]. Periodontitis severity is assessed using radiographs of the alveolar bone loss, the depth of probing (PD), the level of clinical attachment (CAL), and bleeding on probing (BOP). These measures do not track disease activity, illness foci, therapeutic response, or susceptibility to future disease progression. There is still a need for a reliable biomarker to assess periodontitis severity and treatment effectiveness, as well as to recognize and track people who are more likely to develop the condition [4,5]. Identifying biomarkers in progressive periodontal disease is crucial for disease prevention. A gene interaction network consists of genes (nodes) connected by functional edges. These edges are called interactions because the two genes' gene products, such as proteins, may interact physically or affect each other's activity. Studying networks of protein interactions is essential for understanding biological functions because these interactions are necessary for most biological processes. Genes frequently interacting with other genes are hub genes in gene networks. Due to these interactions, hub genes frequently play an essential role in a biological system. GeneMANIA is a versatile and accessible website designed to create theories about gene functionality, examine gene compilations, and determine the priority of genes for practical experiments. Genomic interactions in periodontal disease with a systemic disease like diabetes mellitus have not been explored much.

The development of diagnostic methods for periodontitis is still aided by the integration of salivary biomarker data with outcomes from clinical and radiographic assessments [6]. Salivary biomolecules have the advantage of early, noninvasive host defense and inflammatory event detection that probably takes place before clinical and radiographic evidence of periodontal tissue damage, according to several studies. This biological data helps address the urgent need for more specialized methods to enhance periodontal health [7]. Patients with periodontitis frequently have comorbid systemic conditions like diabetes mellitus, and these conditions may have an impact on the host response parameters and oral health [8]. The Centers for Disease Control and Prevention report that over 30 million adults in the United States are affected by type 2 diabetes mellitus (T2DM). Diabetes can cause changes in salivary composition and poor periodontal health, among other oral manifestations. Protein concentrations found in diabetics' saliva have been found to be altered, which has proven useful for T2DM screening [9]. 

The aim of our research is to determine the localization of chloride intracellular channel 1 (CLIC1) protein as an indicator of the chronic inflammatory condition in the periodontium. The category of metamorphic proteins includes CLIC1 [10,11]. It exists as both an integral membrane protein (tmCLIC1) and a hydrophilic soluble component. CLIC1 is connected to the permeability of chloride ions once in the transmembrane form [12,13]. Recent studies have demonstrated that the enzymes tmCLIC1 and nicotinamide adenine dinucleotide phosphate hydrogen (NADPH) oxidase engage in a feed-forward mechanism, amplifying the production of reactive oxygen species (ROS), particularly within macrophages [14,15]. This process is crucial for expediting the G1/S phase transition during the cell cycle [16]. Furthermore, research has indicated that CLIC1 plays a role in axonal outgrowth [17], supporting the notion that the protein has a functional role related to the cytoskeleton [18,19]. The accumulation of tmCLIC1 protein in chronically activated peripheral blood mononuclear cells, especially monocytes, and the unique localization of CLIC1 may contribute to the proliferation and infiltration of monocytes [20].

The identified hub gene, CLIC1, in the present study as a neoteric marker of systemic inflammation, has not been explored much before. The objective of the present investigation is to ascertain how CLIC1 affects people with and without systemic disease who have periodontitis.

## Materials and methods

The National Center for Biotechnology Information (NCBI) Gene Expression Omnibus (GEO) datasets were used, and data was retrieved for analysis of genes. The online analysis tool named GEO2R (http://www.ncbi.nlm.nih.gov/geo/geo2r) was used to compare the differentially expressed genes between samples. Significantly differentially expressed genes (DEGs) were those with a p-value of 0.05 and a threshold of 1:0 log-fold change. All data is available online. The top 250 significantly differentiated genes commonly involved in periodontal disease and diabetes mellitus were identified. Hub gene networks were constructed using GeneMANIA (University of Toronto, Toronto, Canada), gene ontology analysis was performed using the Enrichr tool (Ma'ayan Laboratory, Icahn School of Medicine at Mount Sinai, NY, USA), and identified hub genes were validated using clinical samples.

The current research was carried out at Saveetha Dental College, Chennai, in the Department of Periodontics. Approval was granted by the Institutional Ethical Committee of Saveetha Dental College (# IHEC/SDC/PERIO-2015/23/202). The patients were divided into three groups according to their clinical and periodontal health. 

There were 30 participants in total, with 16 men and 14 women. Subjects in group 1 (n=10) had good overall health, no gingival inflammation, and probing depths of less than 3 mm. Subjects in group 2 (n=10) were patients who were otherwise healthy but were diagnosed as having stage 2-4 periodontitis based on clinical and radiographic criteria: probing depths of >6mm, interdental clinical attachment loss of 3 to >5 mm in all four quadrants, and radiographic bone loss from the coronal third to the middle third of the root and beyond. Patients in group 3 (n=10) had periodontitis with type 2 diabetes mellitus-glycated hemoglobin values ranging between 6.5 and 7.5.

Participants with type 1 diabetes, smokers, people who had recently used anti-inflammatory drugs, people who had recently undergone periodontal therapy, people with other systemic diseases besides diabetes mellitus like cardiovascular diseases, and expecting mothers or breastfeeding women were excluded from the study.

Each patient signed a written form of informed consent. In the initial appointment, supragingival scaling was performed after assessing the overall periodontal status. After getting an isolated field, saliva was collected by requesting patients to spit inside the Eppendorf tube at the next session early in the morning. Before collection, the subjects washed their mouths with water and waited 15 minutes before pooling saliva. The most effective time to collect saliva was between 9 AM and 12 PM to reduce the diurnal fluctuations brought on by saliva sampling. In order to collect 5 ml of saliva, patients were instructed to sit comfortably and swallow before allowing their saliva to passively drain for about 20 minutes over their lower lip. The presence of cellular debris and the turbidity of saliva can compromise assay accuracy. Thus, to overcome that, all salivary samples were centrifuged at 1000 g (or 3000 rpm) for about 20 minutes. The salivary samples were carefully separated from the supernatant, aliquoted directly into Eppendorf tubes, and stored at a very low temperature of -20 °C to -80 °C.

An enzyme-linked immunosorbent assay (ELISA) reader with 450 nm absorbance measurement capability, automatic plate washer, paper towels, or other absorbent materials, up to 37°C±0.5°C, a constant-temperature incubator that can offer stable incubation conditions, disposable pipettes and tips with single- and multi-channel precision, samples in clean tubes and Eppendorf tubes, distilled or deionized water were used materials for the study.

ELISA measured salivary CLIC1 levels. A human CLIC1 ELISA kit was used. Pre-coated 96-well plates had anti-CLIC1 antibodies. The test was carried out with reagents and samples that had been allowed to reach room temperature (18°-25°C) before introducing the detection antibody, horseradish peroxidase (HRP)-conjugated anti-CLIC-1. The plate, reagents, or samples were not heated in hot water baths. Standard and sample duplicates were on the microplate. 96-well plates were pre-coated with antibodies. The anti-CLIC-1 antibody that was HRP-conjugated served as a reagent. Before commencing the assay procedures, the plate, reagents, and samples were allowed to equilibrate to room temperature, specifically within the range of 18°C to 25°C. It was avoided using hot water baths to heat the plate, reagents, or samples. The microplate contained duplicate standards and samples.

The software used for analyzing the data was SPSS for Windows (version 23.0, IBM Corp., Armonk, NY). To assess the normality of the data, the Shapiro-Wilk test was utilized. For intra-group comparison, the Kruskal-Wallis test was employed, followed by the Mann-Whitney U test for post hoc analysis. 

## Results

Gene network

Gene expression datasets for diabetes and periodontitis were chosen from the GEO database. GEO2R was utilized to locate 250 DEGs from a dataset of diabetes mellitus and periodontitis by comparing them with the pertinent controls present in the datasets. Using the GeneMANIA module of the Cytoscape software 3.1 version (The Cytoscape Consortium, San Diego, CA), an interaction network was created by combining the DEGs from both conditions. Based on functional enrichment analysis, it was shown that the constructed network contained numerous molecular connections that were linked to the DEGs of periodontitis and diabetes mellitus. Thus, the CLIC1 gene was identified as a hub gene in periodontitis in the presence of diabetes mellitus (a systemic disease).

Functional network analysis 

**Figure 1:**
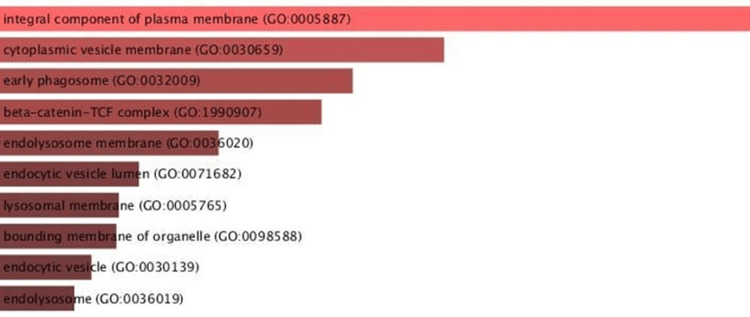
Cellular analysis depicting CLIC1 as an integral component of plasma membrane CLIC1 - chloride intracellular channel 1

The DAVID online tool (DAVID Bioinformatic, Frederick, MD) was used to conduct gene ontology (GO) enrichment analysis to look into the network's functionality. Cellular components, biological events, and molecular functions comprised the three categories of enrichment. The cellular component assessment depicts CLIC1 as an integral component of the plasma membrane, along with others, such as the cytoplasmic vesicle membrane, beta-catenin-T cell factor (TCF) complex, and lysosomal membrane (Figure 1).

**Figure 2:**
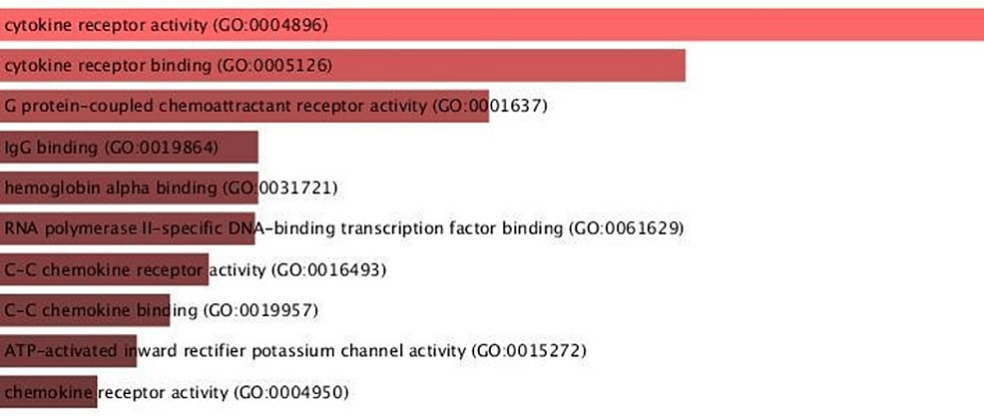
Biological function depicting G protein-coupled chemoattractant receptor activity

The network enrichment analysis, centered on biological events, also unveiled significant associations with cytokine receptor activity, G-protein-coupled chemoattractant receptor activity, hemoglobin alpha binding, and chemokine receptor activity (Figure 2).

**Figure 3:**
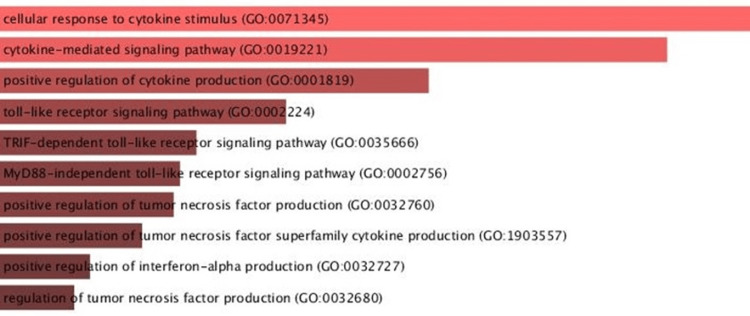
Pathway analysis depicts the role of biomarker in important pathways

Furthermore, a thorough examination of the molecules within the network was conducted to determine their influence on pathway regulation. Notably, these molecules were found to have regulatory roles in pathways such as the cytokine-mediated signaling pathway, the toll-like receptor signaling pathway, the MyD88-independent toll-like receptor signaling pathway, and the regulation of the tumor necrosis factor production pathway (Figure 3).

Using GeneMANIA, the network's genes were simultaneously ranked, showing that CLIC1 was the highest-ranked gene; therefore, it was identified as the hub gene.

The current study's findings show that group 3 had the highest mean salivary CLIC1 concentration, as depicted in Table 1. 

**Table 1:** Statistical difference between the three groups using the post hoc Mann-Whitney test

Group	n	Sum	Average	Variance	p-value
Healthy	10	9.922	0.902	0.0939	0.0245
Periodontitis	10	15.806	1.437	0.7562	0.0268
Periodontitis with diabetes mellitus	10	17.788	1.617	0.2344	0.0355

**Figure 4:**
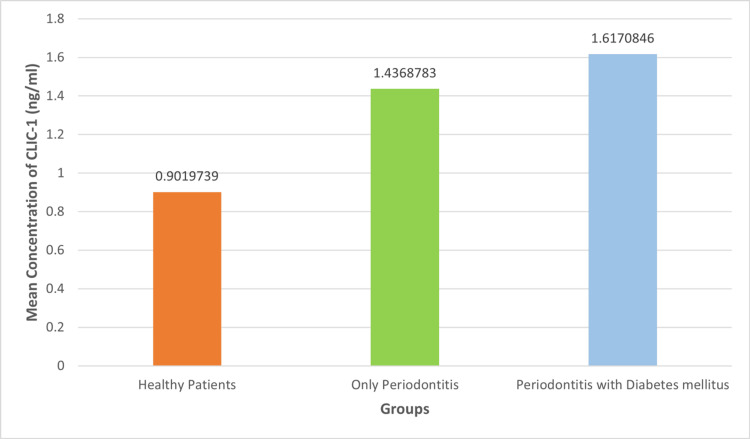
The mean CLIC1 levels in saliva for healthy periodontitis patients and patients of periodontitis with diabetes mellitus CLIC1 - chloride intracellular channel 1

There were 30 participants in the study, aged 25 to 50, of whom 16 were men and 14 were women. The p-value of 0.05 indicated a statistically significant difference in expression among the groups, i.e., healthy vs. only periodontitis (p=0.001), healthy subject vs. periodontitis with diabetes mellitus (p=0.0), and periodontitis only vs. periodontitis with diabetes mellitus (p=0.001). The mean level of CLIC1 (ng/ml) in the healthy group was 0.901; the level in the periodontitis group was 1.436; and the level in the periodontitis with diabetes mellitus group was 1.617. This implies that the mean concentration of CLIC1 rises with disease severity, particularly in patients with systemic comorbidities like diabetes mellitus (Figure 4).

## Discussion

Periodontal disease affects a high percentage of type 2 diabetes mellitus patients, and poorer glycemic control is frequently associated with worsening periodontal conditions [21,22]. This study suggests that more research is necessary to determine the exact mechanism by which periodontal disease and diabetes mellitus are related and that type 2 diabetic patients' periodontal health may depend on how well their diabetes is managed. On the other hand, the management of diabetes in these patients may not be significantly impacted by the treatment of periodontitis. 

According to the results of the current study, group 3, which includes people with periodontitis and diabetes, had a significantly higher mean CLIC1 concentration than group 2, which includes systemically healthy individuals. A p-value of less than 0.005 indicates that there were statistical differences in the expression of CLIC1 between the three groups. This proves that the concentration of salivary CLIC1 consistently increases from a healthy state in periodontitis individuals and is even higher in periodontitis with diabetes mellitus patients. These findings, along with the gene interactome study that identified CLIC1 as the hub gene in periodontitis with systemic disease, implicitly explain why CLIC1 should be used as a diagnostic biomarker for treating periodontitis, especially in patients with systemic disease. 

Saliva is a highly adaptable biological fluid that is simple to gather non-invasively, and the results of its examination assist in completing clinical and histological findings in the diagnosis of various disorders. Building upon the recognition of salivary CLIC1 as a diagnostic biomarker and considering its clinical verification, it was observed that salivary CLIC1 levels were significantly elevated in the periodontitis patient group and notably higher in the group with periodontitis associated with diabetes. Since we found elevated levels of salivary CLIC1 in periodontitis, our results are consistent with earlier research on chronic inflammatory states. 

One of the main cellular compartments where chloride intracellular channels (CLICs) have been identified is the mitochondria, as previously documented [23]. CLICs are known for their ability to influence mitochondrial function by impacting the generation of reactive oxygen species (ROS) and the regulation of calcium levels. It is plausible that CLICs located within mitochondria serve as critical targets for regulating cell proliferation, and modulating their channel activity may prove to be an effective approach for controlling cellular physiology. Mitochondria play a pivotal role in cellular physiology and survival, and recent research has unveiled associations between mitochondrial energetics, dynamics, metabolism, and their involvement in various diseases.

The functional channel formed by CLICs consists of a single transmembrane domain and has been extensively studied using a planar bilayer system, incorporating proteins from bacterial and mammalian species [24]. At least four subunits are required for a channel to function. Occasionally, a mega-chloride (Cl)-channel is formed when separate functional unit subunits come together. Research employing fluorescence resonance energy transfer (FRET) on CLIC1 indicates that when it undergoes oxidation in the presence of membranes, CLIC1 oligomers are composed of 6-8 subunits [25].

A substantial challenge when focusing on CLIC proteins for a specific pathological condition is their widespread presence and their role in the normal physiological processes of cells with various origins. Common features of diseases frequently hinge on the disruption of multiple channels and their associated proteins. Unlike their counterparts in the plasma membrane, intracellular channels generally initiate secondary signaling complexes that are activated by factors within the cytoplasm or from outside the cell. Advancements in the realm of CLICs have ushered in the creation of genetic models and pioneering methodologies for investigating CLICs [26]. These developments will empower researchers to gain a deeper understanding of the specific functions of individual CLICs in various aspects of pathophysiology, encompassing conditions like cancer, hearing impairment, Alzheimer's disease, endocrine disorders, and vascular dysfunction. This investigation could also provide insights into the significance of chloride ions in cellular physiology and potentially pave the way for innovative and unconventional approaches to effectively treating diseases linked to CLICs.

We have established a robust association between periodontitis and T2DM. Our discoveries suggest that it is imperative for dentists to recognize that periodontitis could serve as an indicator for undiagnosed T2DM and suboptimal glycemic control in individuals with T2DM. Physicians should familiarize themselves with the clinical indicators of periodontitis to assist T2DM patients in enhancing their oral hygiene practices and contemplate suggesting periodontal treatment to ameliorate glycemic management. Additionally, patients should be informed that periodontitis and T2DM mutually increase the risk of each other's development. Regular dental care and comprehensive physical examinations are essential for the early prevention of T2DM, or periodontitis.

On the basis of our current knowledge, the study represents the initial effort to identify the salivary biomarker CLIC1 in individuals dealing with both periodontitis and diabetes mellitus. It's worth noting that findings related to the presence of salivary biomarkers in periodontitis may exhibit variances across different research studies. These variations can be attributed to differences in study methodologies, the sizes of the groups being studied, and the dynamic nature of periodontitis as a chronic condition. Also, geographic disparities and ethnic dissimilarities might contribute to differences in data. Beyond the host response and dysbiosis, various factors, such as age, socioeconomic status, smoking, genetics, and geographical location, could influence the condition of periodontal diseases [27]. Therefore, further investigations are imperative to elucidate the potential of any biomarker in association with the diagnosis and progression of periodontitis or as a tool to evaluate the host response to treatments and its correlation with endocrine disorders like diabetes mellitus.

Limitations

This study had several limitations. Firstly, while we managed to identify differences in CLIC1 levels between the groups with diabetic periodontitis, periodontitis alone, and those who were healthy on the basis of statistical analysis, it's probable that the size of the sample was not large enough to accurately assess the genuine variations in CLIC1 levels among the studied population. Additionally, our dataset covered a short time frame and did not include gingivitis or mild periodontitis cases. Consequently, evaluating the connection between levels of biomarkers and the severity of disease posed a significant challenge. These data supplement previous findings and may be used in conjunction with future studies that focus on specific aspects of this subject.

## Conclusions

Within the confines of the study, it is possible to draw the conclusion that CLIC1 is a neoteric biomarker for detecting periodontal disease. To determine CLIC1's role in the etiology of periodontal disease, more research is necessary. In the future, screening large populations for the potential development of destructive periodontal disease may be done using salivary CLIC1 levels. The identification of CLIC-1's role in periodontal inflammation may also lead to the development of novel therapeutic strategies that include host modulation mechanisms.

## References

[1] Seymour GJ (1987). Possible mechanisms involved in the immunoregulation of chronic inflammatory periodontal disease. J Dent Res.

[2] Soundarajan S, Malaippan S, Gajendran PL (2020). Relationship between chief complaints and severity of periodontitis in patients seeking periodontal therapy: a retrospective study. World J Dent.

[3] Radha V, Varghese SS, Ganesh MK (2021). Stability of platelet-rich fibrin treated with tranexamic acid in vivo: a histological study in rats. World J Dent.

[4] Barros SP, Williams R, Offenbacher S, Morelli T (2016). Gingival crevicular fluid as a source of biomarkers for periodontitis. Periodontol 2000.

[5] Korte DL, Kinney J (2016). Personalized medicine: an update of salivary biomarkers for periodontal diseases. Periodontol 2000.

[6] Bell RJ, Rube HT, Kreig A (2015). The transcription factor GABP selectively binds and activates the mutant TERT promoter in cancer. Science.

[7] Verhaak RG, Hoadley KA, Purdom E (2010). Integrated genomic analysis identifies clinically relevant subtypes of glioblastoma characterized by abnormalities in PDGFRA, IDH1, EGFR, and NF1. Cancer Cell.

[8] Mason MR, Preshaw PM, Nagaraja HN, Dabdoub SM, Rahman A, Kumar PS (2015). The subgingival microbiome of clinically healthy current and never smokers. ISME J.

[9] Rao CN, Sood AK, Subrahmanyam KS, Govindaraj A (2009). Graphene: the new two-dimensional nanomaterial. Angew Chem Int Ed Engl.

[10] Harrop SJ, DeMaere MZ, Fairlie WD (2001). Crystal structure of a soluble form of the intracellular chloride ion channel CLIC1 (NCC27) at 1.4-Å resolution. J Biol Chem.

[11] Littler DR, Harrop SJ, Fairlie WD (2004). The intracellular chloride ion channel protein CLIC1 undergoes a redox-controlled structural transition. J Biol Chem.

[12] Valenzuela SM, Mazzanti M, Tonini R (2000). The nuclear chloride ion channel NCC27 is involved in regulation of the cell cycle. J Physiol.

[13] Tonini R, Ferroni A, Valenzuela SM, Warton K, Campbell TJ, Breit SN, Mazzanti M (2000). Functional characterization of the NCC27 nuclear protein in stable transfected CHO-K1 cells. FASEB J.

[14] Milton RH, Abeti R, Averaimo S (2008). CLIC1 function is required for beta-amyloid-induced generation of reactive oxygen species by microglia. J Neurosci.

[15] Ulmasov B, Bruno J, Oshima K (2017). CLIC1 null mice demonstrate a role for CLIC1 in macrophage superoxide production and tissue injury. Physiol Rep.

[16] Peretti M, Raciti FM, Carlini V (2018). Mutual influence of ROS, pH, and CLIC1 membrane protein in the regulation of G(1)-S phase progression in human glioblastoma stem cells. Mol Cancer Ther.

[17] Averaimo S, Gritti M, Barini E, Gasparini L, Mazzanti M (2014). CLIC1 functional expression is required for cAMP-induced neurite elongation in post-natal mouse retinal ganglion cells. J Neurochem.

[18] Kagiali ZCU, Saner N, Akdag M, Sanal E, Degirmenci BS, Mollaoglu G, Ozlu N (2020). CLIC4 and CLIC1 bridge plasma membrane and cortical actin network for a successful cytokinesis. Life Sci Alliance.

[19] Singh H, Cousin MA, Ashley RH (2007). Functional reconstitution of mammalian 'chloride intracellular channels' CLIC1, CLIC4 and CLIC5 reveals differential regulation by cytoskeletal actin. FEBS J.

[20] Cha MH, Rhim T, Kim KH, Jang AS, Paik YK, Park CS (2007). Proteomic identification of macrophage migration-inhibitory factor upon exposure to TiO2 particles. Mol Cell Proteomics.

[21] Mealey BL, Oates TW (2006). Diabetes mellitus and periodontal diseases. J Periodontol.

[22] Borgnakke WS, Glick M, Genco RJ (2013). Periodontitis: the canary in the coal mine. J Am Dent Assoc.

[23] Ponnalagu D, Singh H (2017). Anion channels of mitochondria. Handb Exp Pharmacol.

[24] Gururaja Rao S, Ponnalagu D, Sukur S (2017). Identification and characterization of a bacterial homolog of chloride intracellular channel (CLIC) protein. Sci Rep.

[25] Yang C, Goodchild M, Huang Q (2011). Spatial cloud computing: how can the geospatial sciences use and help shape cloud computing?. Int J Digit Earth.

[26] Singh H, Warburton S, Vondriska TM, Khakh BS (2009). Proteomics to identify proteins interacting with P2X2 ligand-gated cation channels. J Vis Exp.

[27] Chikte U, Pontes CC, Karangwa I, Kimmie-Dhansay F, Erasmus RT, Kengne AP, Matsha TE (2019). Periodontal disease status among adults from south africa-prevalence and effect of smoking. Int J Environ Res Public Health.

